# A single-cell profile of the porcine immune repertoire: concurrent profiling of T cell receptor and B cell receptor V(D)J sequences in health

**DOI:** 10.1007/s44307-026-00139-2

**Published:** 2026-10-09

**Authors:** Hao Zhang, Qixin Zhang, He Zhang, Panfei Wang, Rui Geng, Rufei Pan, Chao Li, Feifei Shi, Jie Liu, Yang Wu, Yongchang Cao

**Affiliations:** 1 State Key Laboratory of Biocontrol, Life Sciences School, Sun Yat-Sen University, Guangzhou, China; 2 State Key Laboratory of Animal Disease Control and Prevention, Harbin Veterinary Research Institute, Chinese Academy of Agricultural Sciences, Harbin, China; 3 Beijing BestopCell Co., Ltd, Beijing, 100176 China

**Keywords:** ScRNA-seq, V(D)J sequencing, Immune repertoire, Pig

## Abstract

Adaptive immune diversity is shaped by the complex organization of T cell receptor (TCR) and B cell receptor (BCR) repertoires across tissues. However, a systematic cross-tissue immune repertoire profiling in healthy pigs is still lacking. In this study, we used 3 14-day-old Duroc × Landrace × Yorkshire (DLY) crossbred piglets, all of which were male. After sample purification, a total of 71,731 cells were retained. We established single-cell profiling integrating paired BCR and TCR repertoires across 11 immune-associated tissues using single-cell RNA sequencing combined with V(D)J sequencing based on DLY crossbred piglets. Diverse populations of immune cells were identified, with significant tissue-specific heterogeneity being revealed in lymphatic, mucosal, and circulatory organs. Immune repertoire analysis demonstrated biased V(D)J gene usage, conserved complementarity-determining region 3 (CDR3) length distributions, preferential receptor pairing patterns, and extensive clonotype diversity. Cross-tissue analyses further revealed coordinated clonotype sharing and distinct clonal expansion patterns among immune cell subsets. In addition, using the Pig-specific V(D)J primers we designed, the sequencing results have achieved a modest improvement in the detection of immunoglobulin alleles compared to previous studies. It is expected to provide a more reliable technical solution for related research. Collectively, this work provides a comprehensive reference profiling of the healthy DLY crossbred piglet adaptive immune system and offers valuable resources for future studies of porcine immunology, infectious diseases, and vaccine responses.

**Supplementary Information:**

The online version contains supplementary material available at https://doi.org/10.1007/s44307-026-00139-2.

## Introduction

Adaptive immunity is essential for host protection from infectious and malignant diseases. It relies on the enormous diversity of TCR and BCR to recognize and eliminate a wide range of pathogens. This diversity is primarily generated through gene segment selection, combinatorial diversity, junctional diversity, and the pairing of heavy and light chains, enabling lymphocytes to establish highly heterogeneous immune repertoires (Chi et al. 2024). The composition and distribution of immune repertoires are closely associated with immune development (Mark et al. 2022), tissue microenvironment (Weisberg et al. 2021), antigen exposure (Baumgartner and Malherbe 2011), and disease status (Wu et al. 2021). Therefore, systematic characterization of TCR and BCR repertoires is essential for understanding immune surveillance, lymphocyte differentiation, and tissue-specific immune regulation (Muñoz-Rojas and Mathis 2021).

As one of the most economically important livestock species worldwide, domestic pigs (*Sus scrofa*) are constantly exposed to a wide range of infectious agents that threaten animal health and agricultural productivity (VanderWaal and Deen 2018; Knox 2025). Three-breed cross pigs, one of the most common breeding varieties in the Chinese countryside, have a more extensive distribution range for farming in China compared to the two-breed cross pigs. They are also threatened by these pathogenic factors, resulting in significant economic losses every year (Zhang et al. 2022; Yan et al. 2023). Precise detection methods and effective prevention and control measures are key to preventing the spread of disease and reducing economic losses (Xia et al. 2024). Most of the prevention and control measures rely on the adaptive immune response of pigs. Effective immune surveillance and adaptive immune responses are therefore critical for disease resistance, vaccine efficacy, and herd health management. The porcine immune system exhibits complex, tissue-specific organization across lymphoid organs, mucosal barriers, and peripheral blood. This organization reflects distinct local immune environments and patterns of antigen exposure (Le Page et al. 2022; Patil et al. 2024). Systematic characterization of TCR and BCR repertoires can provide important insights into immune diversity, clonal dynamics, and host–pathogen interactions (Seo and Choi 2025). However, the overall architecture of porcine adaptive immune repertoires across tissues remains poorly and inaccurately characterized. Previous studies investigating porcine immune repertoires have primarily relied on bulk-sequencing approaches, which provide limited resolution for dissecting cellular heterogeneity and lineage relationships (Butler et al. 2017). Although high-throughput repertoire sequencing has revealed preferential V(D)J gene usage and clonotype expansion patterns in pigs (Chen and Trible 2017; Hammer et al. 2019), these studies generally lack simultaneous transcriptomic information. Therefore, they cannot directly associate receptor features with immune cell identity, functional state, or tissue localization.

Single-cell omics technologies have significantly impacted all areas of modern agricultural research, including plant and animal production (Zhu et al. 2025). Recent advances in single-cell RNA sequencing combined with paired V(D)J sequencing have enabled integrated analysis of gene expression profiles and immune receptor repertoires at single-cell resolution (Pai and Satpathy 2021; Bram et al. 2024). This strategy provides opportunities to investigate lymphocyte heterogeneity, clonal dynamics, and tissue-specific immune organization simultaneously. Nevertheless, comprehensive single-cell profiling integrating both TCR and BCR repertoires across multiple porcine tissues is still scarce. In addition, the baseline immune landscape of healthy pigs remains insufficiently characterized. Most current studies focus on disease models (Yan et al. 2018), vaccine responses (Kekarainen et al. 2010), or specific immune cell populations (Li et al. 2024), whereas systematic investigations of physiological immune repertoire organization across multiple tissues are limited. Establishing a high-resolution reference profiling of healthy porcine immune repertoires is therefore critical for understanding normal immune homeostasis and for providing comparative frameworks for future studies of disease, infection, and immune intervention.

**Fig. 1:**
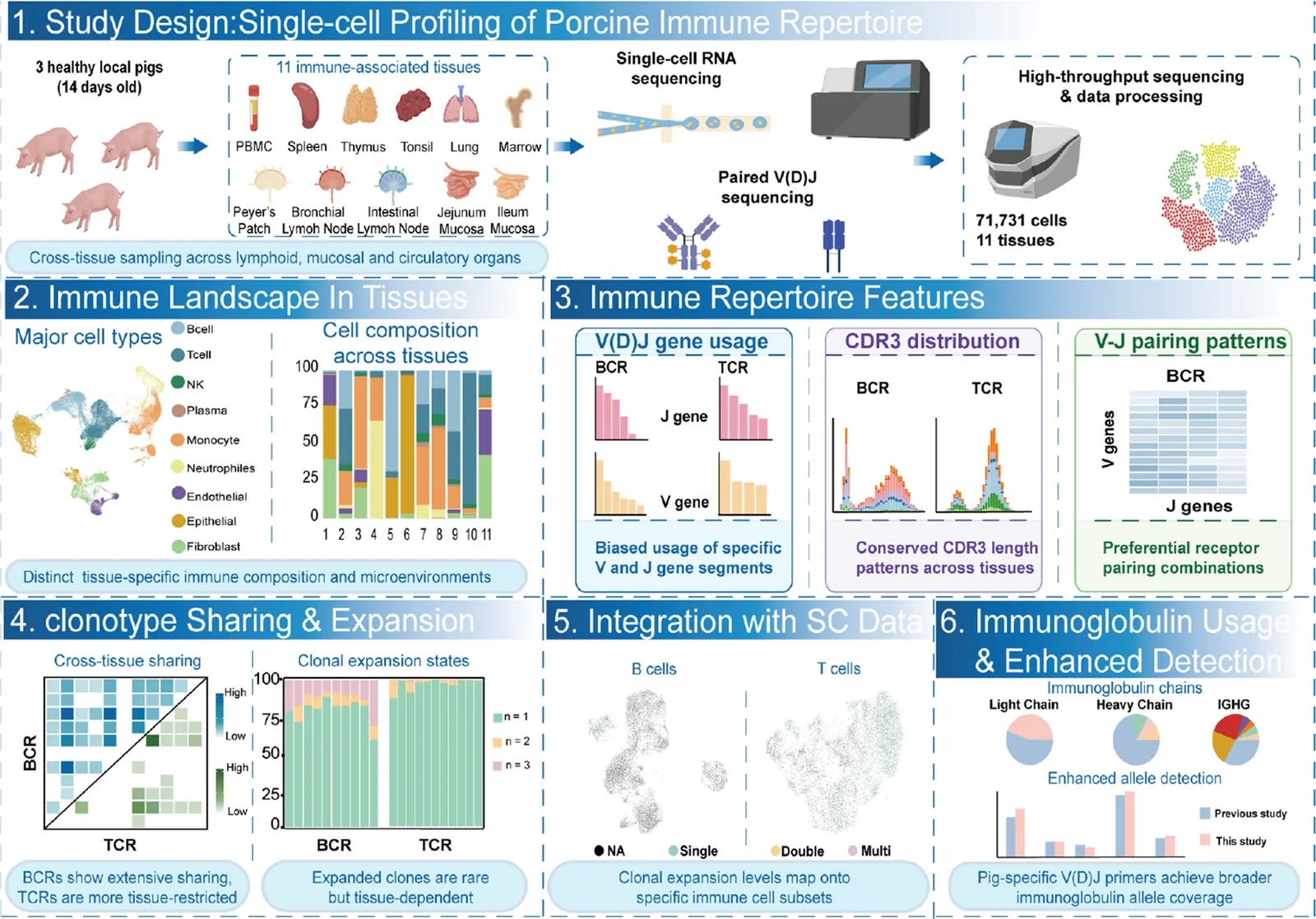
Overview of the experimental design and construction. Immune-associated tissues from healthy 14-day-old pigs were collected and used for sequencing. Comprehensive immune repertoire analyses were performed to investigate V(D)J gene usage, CDR3 length distributions, receptor pairing patterns, and so on. By integrating immune repertoire information with single-cell transcriptomic profiles, the relationships between clonotype dynamics, immune cell differences, and tissue distribution were further explored. The use of pig-specific V(D)J primers enabled improved detection sensitivity and broader immunoglobulin allele coverage. This study provided a valuable reference resource for porcine immunology, infectious disease, and vaccine research

In this study, we designed Pig-specific V(D)J primers and constructed a comprehensive single-cell profiling of the DLY crossbred piglet adaptive immune system by integrating single-cell transcriptomic profiling with paired TCR and BCR repertoire sequencing across 11 immune-associated tissues. To make the research results more applicable, the samples we used were from a mixed sample of 3 healthy pigs. All the samples were mixed according to the sampling location when they were collected. Through large-scale characterization of immune cell populations, we identified extensive heterogeneity among B-cell and T-cell subsets and revealed distinct tissue-specific immune compositions. Moreover, we systematically investigated clonotype diversity, clonal expansion, and cross-tissue clonotype sharing across immune organs. By integrating immune repertoire information with single-cell transcriptomic maps, we further characterized the relationships among clonotype expansion states, cellular differentiation, and immunoglobulin isotype usage. Importantly, through the Pig-specific V(D)J primers we designed, compared with previously reported porcine immune repertoire datasets, the sequencing strategy employed in this study achieved improved detection sensitivity and broader immunoglobulin allele coverage (Gu et al. 2025). The figure shows an overview of the experimental design (Fig. 1). Collectively, this work provides a high-resolution reference profiling of healthy DLY crossbred piglet adaptive immunity and establishes a valuable resource for future studies of porcine immunology, infectious diseases, vaccine responses, and comparative immune biology.

## Materials and methods

### Pigs and sample collection

This study was designed as a descriptive, exploratory atlas based on pooled samples. To construct this atlas, tissue samples from the same anatomical regions were collected from three animals, pooled together prior to cell isolation, and then processed as a single sample per tissue type for subsequent library preparation and sequencing (Wiarda et al. 2026; Zou et al. 2023). Consequently, the sequencing libraries represent pooled biological material rather than individual biological replicates. All samples in the experiment were obtained from the Experimental Animal Center of Guangdong Wens Dahuanong Biotechnology Co., Ltd. The experimental animals were clinically healthy Duroc × Landrace × Yorkshire (DLY) crossbred piglets. All the piglets were male, two weeks old at the time of sampling, and had an average body weight approximately 4.0 kg. The piglets were housed with their mothers after birth and fed with their mothers' milk. The animal rooms were maintained at 22–26 °C with a relative humidity of 50%–70% under a 12 h light/dark cycle. During the rearing period, all piglets underwent routine veterinary examinations and pathogen testing by RT-qPCR for porcine epidemic diarrhea virus (PEDV), transmissible gastroenteritis virus (TGEV), porcine deltacoronavirus (PDCoV), porcine rotavirus (PoRV), porcine reproductive and respiratory syndrome virus (PRRSV), and African swine fever virus (ASFV). Furthermore, to ensure that the baseline immune status of the piglets was not altered, none of the piglets were vaccinated. All pathogen test results were negative. The animals exhibited normal clinical behavior during the rearing period and could therefore be considered healthy and suitable for the study. At the predetermined experimental endpoint, the piglets were humanely euthanized by intravenous administration of sodium pentobarbital (150 mg/kg body weight). Following confirmation of death, necropsy was performed immediately. Within one minute after death was confirmed, whole blood samples were first collected from the jugular vein into EDTA-containing tubes. Peripheral blood mononuclear cells (PBMCs) were subsequently isolated using a porcine PBMC isolation kit according to the manufacturer's instructions. Tissue samples were then collected sequentially in a cranial-to-caudal manner during necropsy.

### Sample preprocessing

Tissue samples from the spleen, lung, thymus, Peyer's patches, tonsils, intestinal lymph nodes, bone marrow, bronchial lymph nodes, ileum mucous membrane, and jejunum mucous membrane were cut into small pieces, washed with RPMI-1640 (Gibco, C11875500137), and stored and transported to the laboratory in Animal Tissue Storage Solution (BestopCell, BA3301). The tissues were subsequently digested using the appropriate dissociation kits. Specifically, the spleen was processed using the Spleen Dissociation Kit (BestopCell, BA3324), the lung was processed using the Lung Dissociation Kit (BestopCell, BA3325), and the thymus, Peyer's patches, tonsils, intestinal lymph nodes, bone marrow, and bronchial lymph nodes were processed using the Multi Tissue Dissociation Kit (BestopCell, BA3303). The ileum and jejunum mucous membrane were processed using the Gastrointestinal Dissociation Kit (BestopCell, BA3316). Tissue dissociation was performed according to the manufacturer's instructions for each kit. At the end of digestion, the cell suspension was passed through a 70 µm nylon cell strainer (Thermo Fisher Scientific, 22–363–548) to remove clumps and debris. The filtrate was then centrifuged at 300 × g for 5 min at 4 °C, after which the supernatant was discarded and the cells were resuspended in phosphate-buffered saline (PBS; Bioss, C7033) containing 5% fetal bovine serum (FBS; Solarbio, S9020). Cell counting revealed the presence of cell debris and dead cells in the suspension. The Dead Cell Debris and Impurities Removal Kit (BestopCell, BA3339) was then used to remove cell debris and dead cells. When only dead cells were present, Dead Cell Removal Solution (BestopCell, BA3338) was used. For digestive system tissues, the corresponding Digestive System Tissue Cell Debris Removal Kit (BestopCell, BA3329) was used. Cells were then counted using a cell counter (Countstar IC1000). Cell counting revealed the presence of red blood cells in the cell suspension. Red Blood Cell Lysis Solution (BestopCell, BA3311) was then used to remove the red blood cells. Following a 15-min incubation at 4℃, the samples were centrifuged at 450 g for 5-min at 4℃. The supernatant was discarded, and the cells were washed twice with PBS containing 5% FBS. Cells were then resuspended in PBS containing 5% FBS. The cells were then counted using a cell counter, and the cell suspension was adjusted to a concentration of 600–1,200 viable cells/µL. PBMCs were isolated from fresh blood samples using PBMC Isolation Solution, pig (BestopCell, BA3364). The procedure was performed according to the manufacturer's instructions. Subsequent processing was performed as described above for the other tissues.

### Library construction and sequencing

Single-cell capture was performed using the CelCode® High-Throughput Single Cell 5’ transcriptome Kit (BestopCell, BA3111) according to the manufacturer's instructions. Briefly, a total of 70 µl of master mix and cell suspension containing 17,000 cells, 70 µl of barcoded gel beads, and 270 µl of partitioning oil were loaded into the CelCode® Driver to generate gel beads-in-emulsion (GEMs). Immediately following GEM generation, the gel beads were dissolved and primers were released. The polyadenylated mRNA was reverse-transcribed into cDNA. The resulting cDNA was amplified, and its quality was assessed using an Agilent 2100 Bioanalyzer. Full-length cDNA containing cell barcode informations was PCR-amplified, and sequencing libraries were subsequently prepared and normalized. The libraries were finally sequenced on a MGI DNBSEQ-T7 sequencer at a depth of at least 20,000 reads per cell using paired-end 150-bp (PE150) sequencing by BestopCell (Beijing, China).

Pig-specific V(D)J primers were designed based on the manufacturer's guidelines. The Pig Single-Cell TCR Amplification Kit (BestopCell, BA3121) and Pig Single-Cell BCR Amplification Kit (BestopCell, BA3122) were used to amplify and enrich TCR and BCR sequences, respectively. The CelCode® High-Throughput Single-Cell Immune Repertoire Library Kit (BestopCell, BA3120) was subsequently used to construct the TCR and BCR libraries. The libraries were finally sequenced on a MGI DNBSEQ-T7 sequencer at a depth of at least 5,000 reads per cell using paired-end 150-bp (PE150) sequencing by BestopCell (Beijing, China).

Raw sequencing reads were demultiplexed and mapped to the reference genome using the CelScene® pipeline with default parameters. Alignment, filtering, barcode counting, and UMI counting were performed with the CelScene® count module to generate feature-barcode matrices.

### Data analysis

The reference genome used for sequence alignment was Sscrofa11.1. The resulting gene-barcode matrices were loaded into R version 4.4.1 for downstream analysis. Clustering analyses were performed using Seurat v4.4.0 (Stuart et al. 2019). Low-quality cells were excluded according to the following criteria: the number of detected genes was outside the range of 200 to 5,000; (ii) the total UMI count was outside the range of 1,000 to 25,000; (iii) the mitochondrial gene expression exceeded the threshold specified in Supplementary Material 6. Additionally, genes detected in fewer than 3 cells were excluded from further analysis. Subsequently, the samples were integrated using a standard integration workflow. Data were normalized using SCTransform (Hafemeister and Satija 2019), and variable features were identified accordingly. Samples were then integrated using CCA (Butler et al. 2018). To assess the effectiveness of batch correction, we quantified batch mixing and cell-type conservation using the integration Local Inverse Simpson’s Index (iLISI) and cell-type Local Inverse Simpson’s Index (cLISI), respectively. Following integration, the iLISI increased from 1.407 to 1.815, indicating improved mixing among cells from different batches. In contrast, the cLISI showed minimal change, decreasing from 1.092 to 1.080, suggesting that the integration effectively reduced batch effects while largely preserving cell-type-specific structure (FigS. 1 C). Following integration, dimensionality reduction and clustering were performed using RunPCA, FindNeighbors, FindClusters, and RunUMAP. Cell clusters were annotated based on the expression of canonical marker genes reported in the literature and curated in databases such as CellMarker and PanglaoDB (Zhang et al. 2018; Franzen et al. 2019). Marker genes were identified using the FindAllMarkers function with the Wilcoxon rank-sum test, applying the following thresholds: log2 fold change (log2 FC) > 0.5, a minimum percentage of 25% of cells expressing the gene, and adjusted *p*-value < 0.05 after Benjamini–Hochberg correction.

The scRepertoire v2.2.0 (Yang et al. 2025) was used to track clonotypes across samples and integrate V(D)J repertoire data with single-cell transcriptomic data. The loadContigs function was used to generate and format the contig data. Subsequently, the combineBCR and combineTCR functions were used to combine BCR and TCR sequencing data at the individual cell-barcode level. Functions including percentGeneUsage, clonalLength, and clonalAbundance were subsequently used to analyze V and J gene usage and CDR3 lengths of BCR and TCR sequences. Finally, the combineExpression function was used to integrate immune repertoire data with single-cell transcriptomic data based on matched cell barcodes. Subsequent analyses were performed following the standard workflow. Figures were generated using R version 4.4.1 and subsequently formatted using Adobe Illustrator 2024.

## Results

### Single-cell RNA sequencing analysis of tissues from healthy pigs

**Fig. 2:**
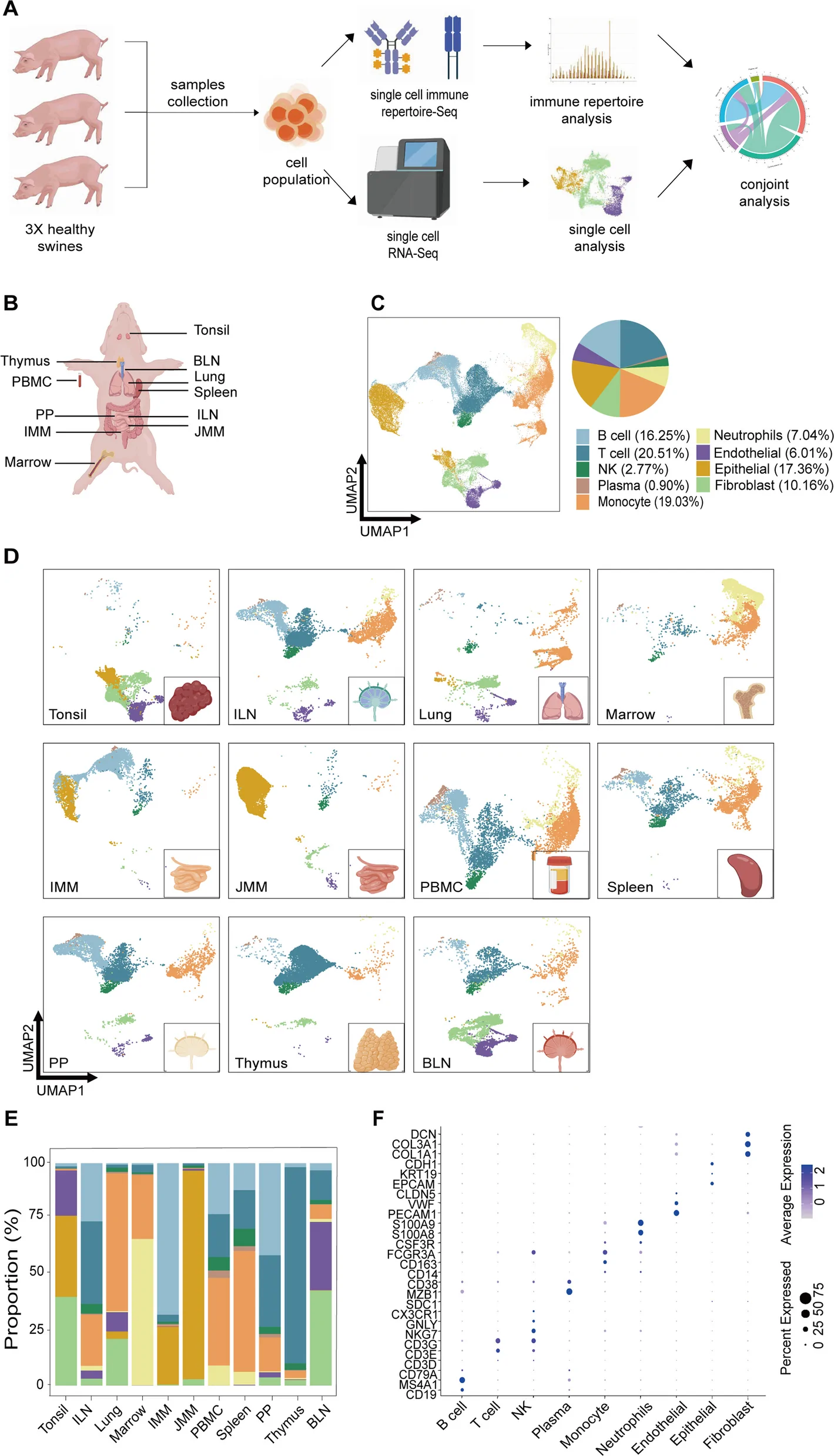
Construction of a single-cell profiling across multiple porcine tissues (ILN: Intestinal Lymph Node; IMM: Ileum Mucous Membrane; JMM: Jejunum Mucous Membrane; PBMC: Peripheral Blood; PP: Peyer’s Patch; BLN: Bronchial Lymph Node). **A** Schematic overview of the experimental workflow. Immune-associated tissues from healthy pigs (14 days) were collected for single-cell RNA sequencing and paired TCR/BCR repertoire sequencing. **B** Anatomical illustration of the 11 sampled tissues. **C** UMAP visualization of integrated single-cell transcriptomes grouped by tissue origin. **D** Global UMAP projection of all cells colored by annotated cell types. **E** Proportions of major cell populations across different tissues.** F** Bubble plot showing expression patterns of marker genes used for cell-type annotation

To comprehensively characterize the porcine immune system, we performed integrated single-cell RNA sequencing and paired immune repertoire sequencing across 11 tissues from 3 healthy pigs at 14 days of age (Fig. 2A). The tissues sampled span major lymphoid, mucosal, circulatory, and immune-associated organs, including peripheral blood, spleen, thymus, tonsil, lung, bone marrow, intestinal lymph node, bronchial lymph node, Peyer’s patch, and intestinal mucosal tissues (Fig. 2B) (Jiang et al. 2025). This multi-organ sampling strategy enabled a systematic investigation of tissue-specific immune heterogeneity and lymphocyte distribution patterns in healthy pigs.

After quality control (FigS. 1 A) and removal of red blood cell contamination, we deleted a total of 22,141 low-quality cells (FigS. 1B) and obtained 71,731 cells in total. Dimensionality reduction analysis identified multiple transcriptionally distinct cell populations across all tissues (Fig. 2C). Major cell types include T cells, B cells, NK cells, plasma cells, monocytes, neutrophils, endothelial cells, epithelial cells, and fibroblasts. Uniform Manifold Approximation and Projection (UMAP) visualization of individual tissues further revealed distinct cellular composition patterns and tissue-dependent immune microenvironments (Fig. 2D). Lymphoid tissues such as spleen, lymph nodes, and tonsil were enriched for adaptive immune cells. In contrast, mucosal and lung-associated tissues exhibited increased structural and innate immune cell components. Cell-type annotation was validated using canonical marker genes (Fig. 2F). B cells highly expressed *CD19*, *MS4A1*, and *CD79A*, while T cells expressed *CD3D*, *CD3E*, and *CD3G*. *NKG7* and *GNLY* characterized NK cells, whereas plasma cells showed elevated expression of *MZB1* and *CD38*. Monocyte and neutrophil populations were identified by expression of *CD14*, *FCGR3A*, *S100A8*, and *S100A9*. Structural cell populations, including endothelial, epithelial, and fibroblast, displayed expected lineage-specific markers, including *PECAM1*, *VWF*, *EPCAM*, and *COL1A1*.

Comparative analysis of cellular composition across tissues demonstrated marked heterogeneity in immune cell distribution (Fig. 2E). Adaptive immune cells predominated in lymphoid organs, whereas epithelial and stromal populations were more abundant in mucosal tissues. B cells showed tissue-selective enrichment in gut-associated immune tissues, suggesting active humoral immune functions within mucosal environments.

### Heterogeneity and tissue distribution of porcine B- and T-cell subsets

**Fig. 3:**
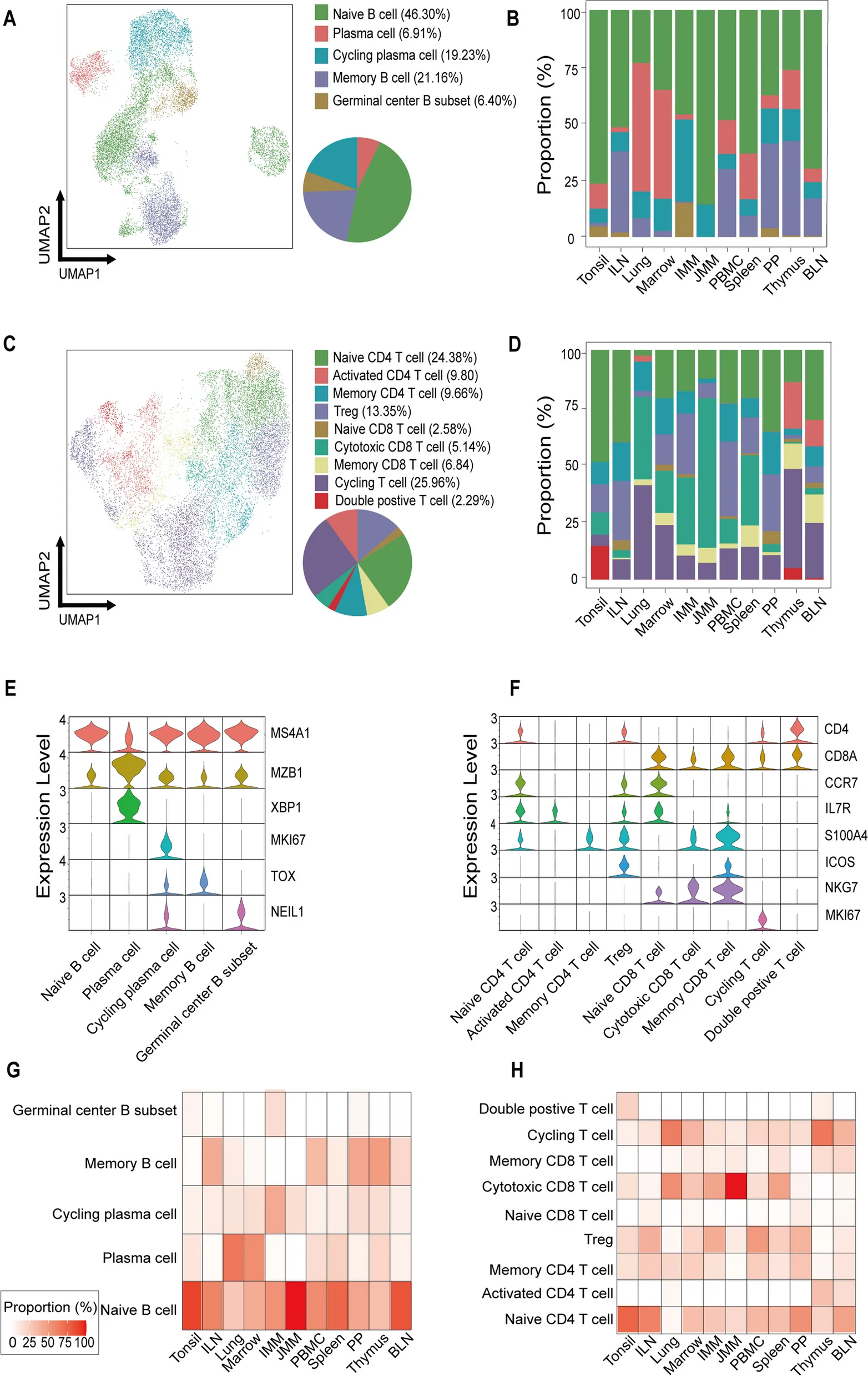
Reclustering and tissue distribution of porcine immune cell subsets. **A** UMAP visualization of B-cell populations. **B** Proportions of B-cell subsets across different tissues. **C** UMAP visualization of T-cell populations. **D** Proportions of T-cell subsets across different tissues. **E–F** Violin plots showing expression of marker genes across B-cell and T-cell subsets. **G-H** B-cell and T-cell subsets are presented with normalized enrichment patterns in heatmaps

The re-annotation of cell subpopulations is indispensable for further analysis of the immune heterogeneity of the pig body, which can further enhance the resolution of cell clusters and better distinguish similar cells. This is also a commonly used method in other studies (Thai et al. 2026). B and T cells were separately reclustered and annotated using lineage-specific transcriptional features. Reclustering of B cells identified five major subpopulations, including naive B cells, memory B cells, germinal center B subsets, plasma cells, and cycling plasma cells (Fig. 3A). These subsets exhibited distinct transcriptional states associated with B-cell activation, differentiation, and antibody production. Cell-type identities were validated using canonical marker genes (Fig. 3E). Naïve B cells showed elevated expression of *MS4A1*, whereas plasma cell populations highly expressed *MZB1* and *XBP1*. Cycling plasma cells additionally displayed strong *MKI67* expression, indicating active proliferation. Germinal center B subsets and memory B cells exhibited intermediate activation-associated transcriptional signatures, which respectively express relatively strong *NEIL1* and *TOX*.

T-cell reclustering resolved nine transcriptionally distinct subpopulations, including naive CD4 T cells, activated CD4 T cells, memory CD4 T cells, Treg cells, naive CD8 T cells, cytotoxic CD8 T cells, memory CD8 T cells, cycling T cells, and double-positive T cells (Fig. 3C). These populations reflected diverse functional and developmental states within the porcine T-cell compartment. Marker gene analysis further confirmed T-cell annotation accuracy (Fig. 3F). Naive T-cell subsets expressed *CCR7*, whereas cytotoxic CD8 T cells were characterized by high *NKG7* expression. Treg possesses high *ICOS* expression. Activated and memory T-cell populations displayed increased *IL7R* and *S100A4* expression. Cycling T cells showed elevated *MKI67*, consistent with proliferative activity, while double-positive T cells simultaneously expressed *CD4* and *CD8A*.

Comparative analysis of tissue composition revealed substantial heterogeneity in the distribution of adaptive immune subsets across organs. Naive B cells represented the dominant B-cell population in most tissues. In the meantime, plasma cells and cycling plasma cells were preferentially enriched in mucosal and lymphoid tissues (Fig. 3B). Germinal center B subsets displayed tissue-restricted enrichment patterns, suggesting localized humoral immune activation. Similarly, T-cell subset composition varied markedly among tissues (Fig. 3D). Naive T-cell populations were highly abundant in lymphoid tissues, while cytotoxic and memory CD8 T cells were enriched in peripheral and mucosal tissues. Double-positive T cells were predominantly observed in thymic-associated compartments, consistent with thymocyte developmental characteristics. Heatmap analysis further highlighted tissue-specific enrichment patterns of B-cell and T-cell subsets (Fig. 3G, H). Distinct adaptive immune architectures were observed across organs, reflecting functional specialization of local immune microenvironments. Together, these results reveal extensive cellular heterogeneity and spatial organization within the porcine adaptive immune system.

### Immune repertoire characteristics of porcine BCR and TCR sequences

**Fig. 4:**
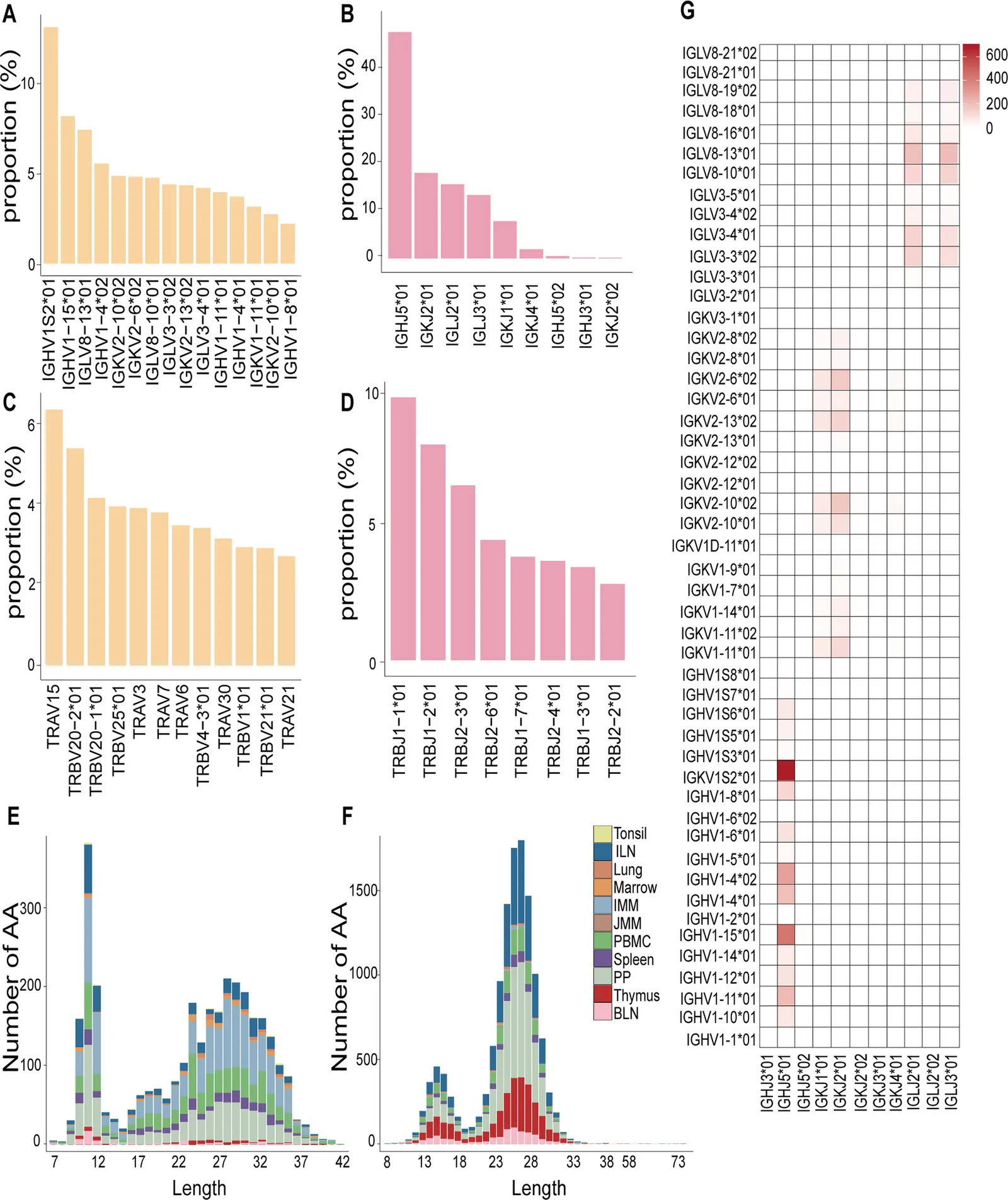
Characteristics of porcine BCR and TCR repertoires across tissues. **A-B** Frequency of BCR V genes and J genes across all tissues (partial). **C**-**D** Frequency of TCR V genes and J genes (partial). **E–F** CDR3 amino acid length (heavy chain and light chain) distributions of BCR and TCR chains separated by different tissues. **G** BCR V–J gene pairing frequencies are shown in a heatmap

By using the high-throughput single-cell immune repertoire sequencing method, we identified a total of 3,275 BCR clones and 13,431 TCR clones from 11 tissue samples obtained from pigs. In the process of characterizing immune repertoire features across porcine tissues, we systematically analyzed V(D)J gene usage patterns and CDR3 length distributions of paired BCR and TCR sequences. BCR repertoire analysis revealed biased usage of specific immunoglobulin V genes, with several *IGHV*, *IGKV*, and *IGLV* families exhibiting dominant frequencies across tissues (Fig. 4A) (Zhang et al. 2025; Partners 2026). For example, the top five BCR V genes—IGHV1S2*01, IGHV1-15*01, IGLV8-13*01, IGHV1-4*02, and IGKV2-10*02—together accounted for 39.41% of total V-gene usage. Similarly, J-gene usage was also skewed toward specific segments, with IGHJ5*01 alone representing 44.95% of J-gene usage. These observations point to non-random recombination patterns in the porcine B-cell repertoire. (Fig. 4B).

Analysis of TCR repertoires also demonstrated selective V and J gene utilization. Multiple *TRAV* and *TRBV* segments displayed markedly elevated frequencies, for example, *TRAV15* (6.41%) and TRBV20 − 2*01 (5.44%), suggesting conserved or preferential TCR recombination features in healthy pigs (Fig. 4C). Correspondingly, distinct *TRAJ* and *TRBJ* gene segments were preferentially enriched within the T-cell repertoire (Fig. 4D). Such as TRBJ1 − 1*01 (9.63%), TRBJ1 − 2*01 (7.90%) and TRBJ2 − 3*01 (6.42%). Together, these results indicate highly structured and biased V(D)J gene usage in both BCR and TCR repertoires. More detailed statistics on the usage frequencies of V genes and J genes of BCR and TCR can be found in this figure (FigS. 2A-H).

We next examined the length distribution of BCR and TCR CDR3 sequences across different tissues. Distinctive length distribution patterns were observed for the CDR3 regions of different clonotypes. Specifically, the CDR3 lengths of BCR were predominantly enriched within the amino acid intervals of 10–12 and 29–31 residues (Fig. 4E), whereas TCR CDR3 lengths were mainly concentrated within the 12–14 and 26–28 residue ranges (Fig. 4F). In both receptor types, CDR3 lengths were restricted to relatively narrow amino acid intervals, reflecting the conserved structural constraints imposed during antigen receptor generation. Moreover, the patterns of CDR3 length variation remained relatively stable across tissues, further indicating the overall structural conservation of adaptive immune receptors among immune-related organs. It confirms the relevant reports (Mandric et al. 2020).

To further investigate BCR and TCR recombination characteristics, we analyzed V–J pairing frequencies across immunoglobulin chains. In BCR, distinct V–J combinations showed preferential enrichment (Fig. 4G). For example, the most prominent ones -in the heavy chain segment combinations—include *IGHV1S201-IGHJ501*, *IGHV1 − 1501-IGHJ501*, and *IGHV1 − 402-IGHJ501*; while in the light chain segment combinations, the notable ones are *IGLV8 − 1301-IGLJ201* and *IGKV2 − 1002-IGKJ201*. In TCR, the frequency distribution of the segment combinations of the α and β chains is relatively more uniform (FigS. 3 A,B). The combination characteristics are manifested in the frequent use of V genes such as *TRAV7*, *TRAV15*, and TRBV20-2*01, as well as J genes such as TRAJ43*01, TRAJ47*01, and TRBJ1-1*01.

Collectively, these results demonstrate that porcine adaptive immune repertoires exhibit structured V(D)J gene usage, conserved CDR3 length distributions, and preferential receptor pairing patterns, providing a comprehensive overview of baseline immune repertoire architecture in healthy pigs.

### Clonal diversity, expansion, and cross-tissue sharing of porcine immune repertoires

**Fig. 5:**
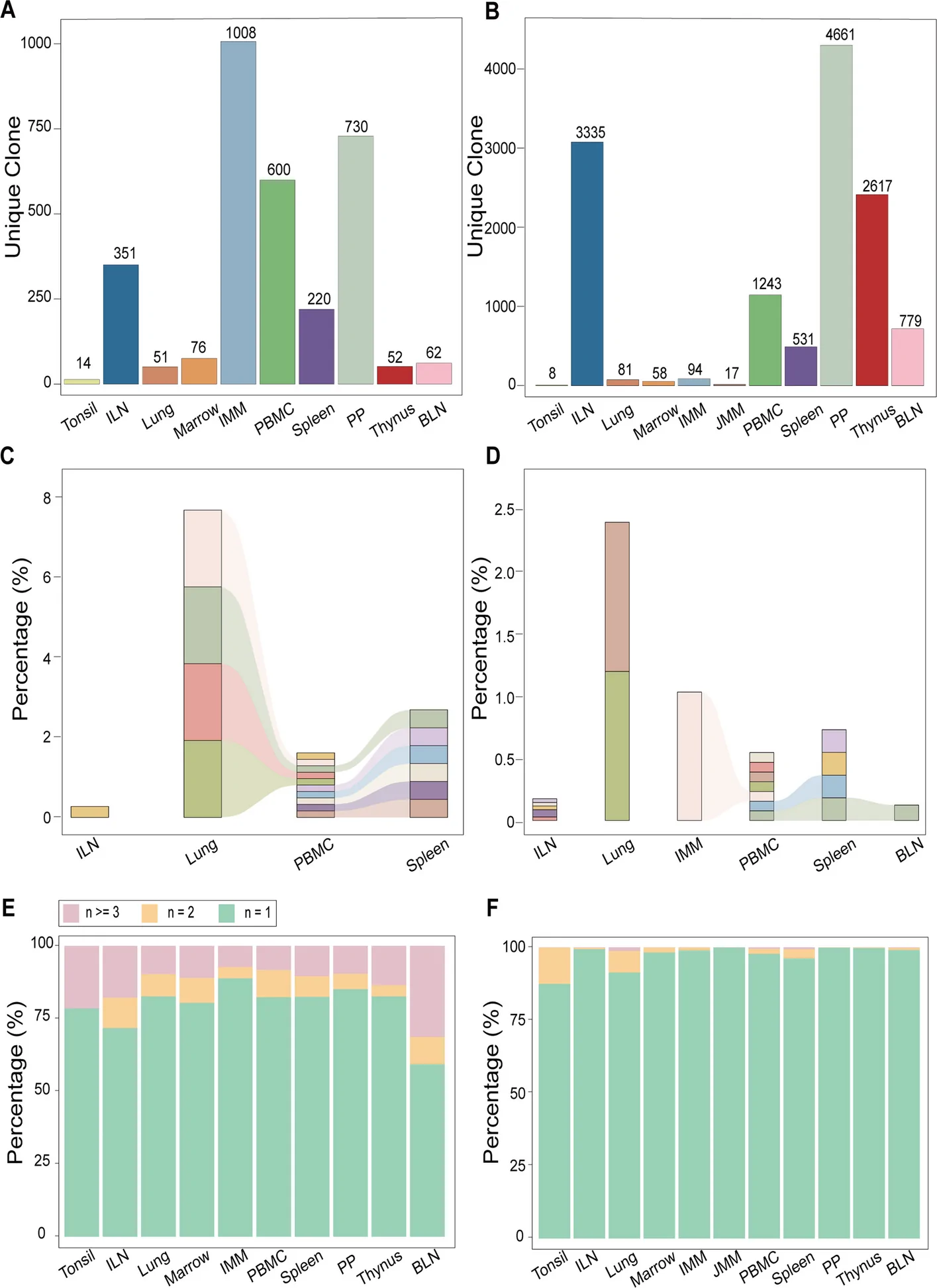
The distribution of unique clonotypes, major clonotypes and clonal states. **A-B** Numbers of unique BCR and TCR clonotypes in each tissue. **C**-**D** Cross-tissue distribution of the top 10 complete BCR and TCR clonotypes. **E–F** Distribution of clonotype expansion categories across tissues in BCR and TCR repertoires; *n* means the number of this clonotype detected in all tissues

Clonotype is the basis of immune repertoire analysis. In subsequent research, a unique clonotype is defined as a clonotype with identical CDR3 amino acid sequences in the heavy and light chains or the α and β chains. For the few incomplete chains, based on the consideration of completeness from the experimental results, we decided to retain them. We first quantified unique clonotypes within BCR and TCR repertoires. Substantial variation in clonotype diversity was observed among tissues, with peripheral blood, Peyer’s Patch, and intestinal lymph node generally harboring larger numbers of unique clonotypes than other tissues (Fig. 5A, B). Clonotype abundance analysis further demonstrated that most clonotypes were present at low frequencies in both BCR and TCR repertoires, whereas highly expanded clonotypes accounted for only a limited fraction of total clones (FigS. 4 A, B). This distribution pattern indicates that porcine immune repertoires maintain high baseline diversity under physiological conditions.

We then screened cells with intact receptor structures (intact receptors with both light and heavy chains). The distribution of complete and dominant clonotypes across tissues was examined. Multiple top clonotypes are distributed across various tissues, including CARGRIAMVLVAMMSSMDLW_CQQFKEFPPGF, CAARSGGNYRPIF_CASSRDLQTQYF, CAVRNNNDLRF_CAWSLYRGVTEVFF, CARGIMAIAMVLVAMPYYAMDLW_CQSADSSDVPF, CAAMSYNKLMF_CASSGTGDIQYF, CAAPSGTGWQVIF_CASSIHRDYEVFF, and so on. Analysis of the top 10 clonotypes revealed extensive inter-tissue sharing in both BCR and TCR repertoires (Fig. 5C, D). Several clonotypes were broadly distributed across multiple immune-associated tissues. Especially in PBMC, the variety of shared receptors is the highest, suggesting active lymphocyte recirculation via blood circulation.

To further characterize clonal expansion states, clonotypes were stratified into three categories by clone size: single clonotypes (*n* = 1), double clonotypes (*n* = 2), and multiple clonotypes (n ≥ 3). Across both BCR and TCR repertoires, single clonotypes were the predominant fraction in most tissues, indicating extensive repertoire heterogeneity (Fig. 5E, F). Compared with TCR repertoires, BCR repertoires showed greater inter-tissue variability in expanded clone proportions, suggesting a distinct difference of organizational dynamics between humoral and cellular immunity. We further assessed repertoire diversity using multiple diversity indices (Mika et al. 2025), including Shannon (Hill 1973), inverse Simpson, ACE (Chao and Lee 1992), and Chao1 (Chao 1984) scores (FigS. 4 C, D). Within the BCR repertoire, evenness is highly conserved across most tissue groups (with a narrow peak near 2.6 for Shannon), in contrast to richness, which exhibits extreme heterogeneity. Tissues represented by the bone marrow and thymus exhibit significantly higher abundance than other tissues. Similarly, most tissues display nearly identical high evenness (approximately 2.07 for Shannon) and different richness within the TCR repertoire. Overall, these findings demonstrate clonal heterogeneity and homogeneity among different tissues in pigs and preliminarily depict the cross-tissue coordination ability of the porcine immune system.

### Clone mapping and clonotype overlap analysis

**Fig. 6:**
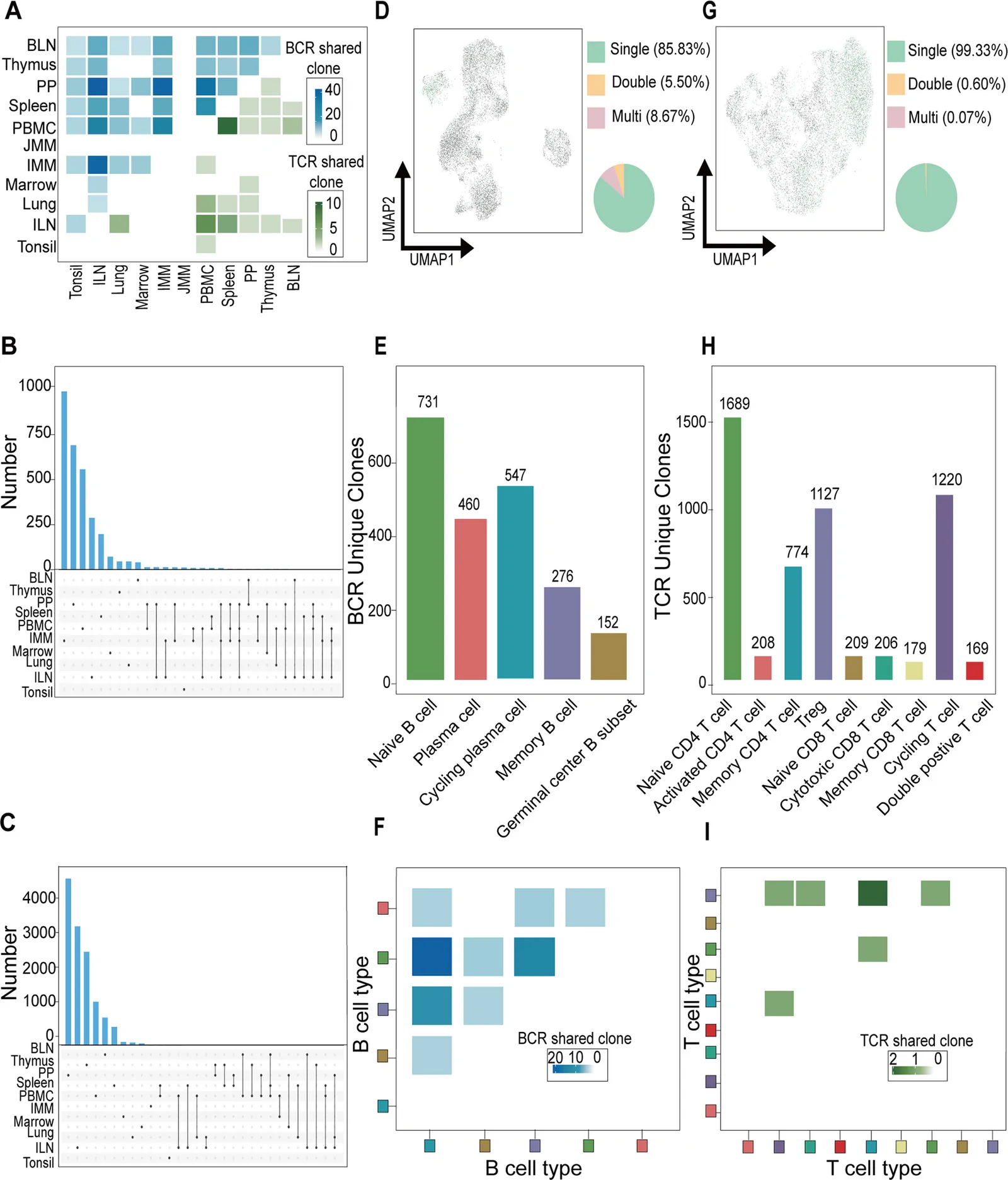
Cross-tissue clonotype sharing and clonal expansion patterns of immune cell subsets. **A** Number of shared clonotypes between tissues are shown in a heatmap. The upper-left matrix represents BCR clonotype overlap, while the lower-right matrix represents TCR clonotype overlap. **B-C** The top 30 shared clonotype combinations among tissues for BCR and TCR repertoires after within-tissue clonotype deduplication are shown in UpSet plots. **D** UMAP visualization of B cells colored according to clonotype expansion level. **E** Numbers of unique clonotypes within B-cell subsets. **F** Numbers of shared clonotypes among B-cell subsets. **G** T cells are colored according to clonotype expansion level. **H** Numbers of unique clonotypes within T-cell subsets. **I** Numbers of shared clonotypes among T-cell subsets

An extended analysis of immune connectivity between tissues remains to be done. We analyzed clonotype sharing patterns across organs (Fig. 6A). The majority of BCR clone-sharing organs include the Peyer's Patch, intestinal lymph node, ileum mucous membrane, and peripheral blood. In contrast, TCR clones show higher specificity, with few cases of clonal sharing among different organs. The tissues with the most shared clones are the peripheral blood and spleen (11 shared clones). Subsequently, to minimize the influence of clonal expansion bias and accurately count overlapping unique clones, clonotypes within each tissue were deduplicated prior to overlap analysis. UpSet analysis of the top 30 shared clonotype combinations demonstrated the unique patterns of clonotype sharing across tissues (Fig. 6B, C). The majority of clonotypes exhibit a distinct tissue-specific distribution, while the rest are present in two or more tissues simultaneously, reflecting the coexistence of local adaptability and the circulatory nature of the immune system.

Then, we mapped the repertoire information to the single-cell transcriptomic map through one-to-one corresponding cell barcodes, obtaining a dimension-reduced visualization map of cells containing BCR and TCR information. After performing the mapping operation based on cell barcodes, a total of 2237 clones were mapped to the B-cell map, and 5786 to the T-cell map. UMAP visualization of B cells showed clear associations among cell subtype identity and clonotype expansion level (Fig. 6D). The B cell receptors are conspicuously concentrated in the plasma cell and cycling plasma cell subgroups. In terms of quantity, the naïve B cell population has the largest number of clones (Fig. 6E). Among T cell subsets, the T cell receptors are concentrated in the naive CD4 T cells, naive CD8 T cells, Treg cells, and memory CD4 T cells (Fig. 6G). The number of receptors was the highest in the naive CD4 T cells, cycling T cells, and Treg subgroups (Fig. 6H). Based on the shared clone heatmap of different cell subpopulations, we found that BCR clones were mainly shared among naïve B cells, memory B cells, and cycling plasma cells (Fig. 6F). TCR clones were mostly present in a monoclonal form, and the occurrence of shared clones was rare (Fig. 6I). These results reveal the cross-tissue sharing and specific expansion patterns of the immune system, highlighting the specialized characteristics of immune cells.

### Clonal expansion and immunoglobulin isotype features of porcine immune cells

**Fig. 7:**
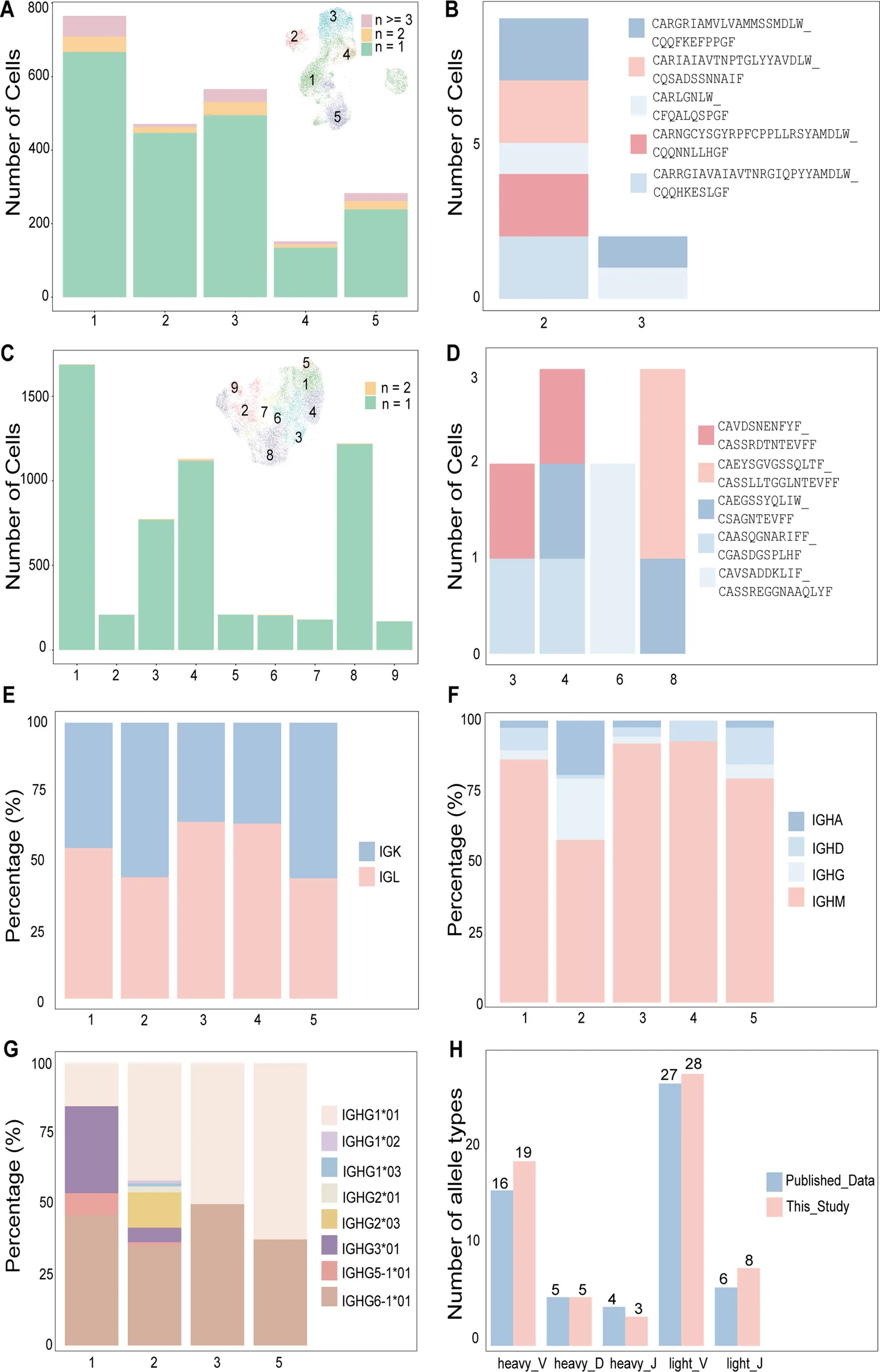
Clonal expansion and immunoglobulin isotype features. **A** Distribution of BCR clonotype expansion categories across B-cell subsets. **B** Distribution of the top 5 complete and dominant BCR clonotypes. **C** Distribution of TCR clonotype expansion categories across T-cell subsets. **D** Distribution of the top 5 complete and dominant TCR clonotypes. **E** Proportions of IGK and IGL light-chain usage. **F** Proportions of immunoglobulin heavy-chain isotypes. **G** Distribution of IGHG subclass alleles. **H** Comparison of the numbers of detected immunoglobulin allele types between this study and a published reference dataset

To investigate the relationship between clonal expansion and immune cell differentiation, we analyzed clonotype expansion states across B-cell and T-cell subsets. In the BCR, the single clonotype (*n* = 1) predominates in each B cell subset, and the high-expansion clonotypes (n > = 2) are relatively more abundant in the cluster 1 and 3 (Fig. 7A). The distribution statistics for the top 5 intact clones revealed that cluster 2 had a significant advantage in the number of shared clones (Fig. 7B). In the TCR, the number of high-expansion clonotypes is very limited, and the only high-expansion clonotypes are mainly concentrated in the cluster 4 (Fig. 7C). This is also confirmed by the top 5 clone distribution chart (Fig. 7D). These statistical results reveal the wide distribution of single clonotypes and the cell type preference of high-expansion clonotypes in the process of enrichment.

We then carried out the separation and statistics of the immunoglobulin chains, intending to conduct a more in-depth study on the usage of immunoglobulins. Analysis of light-chain composition revealed slightly different usage patterns of Immunoglobulin Kappa (IGK) and Immunoglobulin Lambda (IGL) (Fig. 7E). Cluster 1, 3, and 4 use IGL more frequently, while on Cluster 2 and 5, IGK has a greater advantage, suggesting differential light-chain selection during B-cell maturation and activation. Heavy-chain isotype analysis demonstrated substantial variation in Immunoglobulin Heavy Constant Alpha (IGHA), Immunoglobulin Heavy Constant Delta (IGHD), Immunoglobulin Heavy Constant Gamma (IGHG), and Immunoglobulin Heavy Constant Mu (IGHM) usage (Fig. 7F). IGHM accounts for more than 50% in each cluster. IGHA and IGHG have a higher proportion in cluster 2, while the distribution of IGHD is more evenly distributed. As the most common type of antibody, we conducted further allele statistics on IgG (Fig. 7G). IGHG1*01 and IGHG6 − 1*01 were significantly enriched in all clusters, and in cluster 2, the types of IGHG alleles were the most diverse. Variations in the composition of B cell receptors underlie functional heterogeneity among B cell populations (Hobeika et al. 2016; Graf et al. 2019). In our study, distinct B cell subpopulations exhibit characteristic differences in immunoglobulin heavy chain repertoire composition. Notably, the plasma cell subpopulation displays a significantly enriched representation of IGHA and IGHG encoded heavy chains relative to other B cell subsets. Moreover, the diversity of IGHG subclass usage is markedly elevated in this population. It mirrors the diversity of the BCR structure composition and its preference for certain structural components. In the present study, we focus primarily on the comprehensive characterization of immunoglobulin subtype enrichment and clonal sharing patterns across tissues. The underlying transcriptional regulatory network mechanism remains to be further explored through future related research.

To evaluate sequencing performance, we compared the number of identified immunoglobulin allele types with those reported in a published reference dataset. Our study detected substantially more heavy-chain V, light-chain V and light-chain J alleles across immune repertoires (Fig. 7H), demonstrating markedly improved resolution and sensitivity in immune repertoire characterization. These results indicate that the sequencing strategy used in this study provides enhanced coverage and more comprehensive detection of porcine immunoglobulin diversity compared with previously reported approaches.

## Discussion

Adaptive immune diversity is primarily generated through the stochastic recombination of V, D, and J gene segments, together with junctional diversification, nucleotide insertion or deletion, and receptor chain pairing (Roth 2014). Following antigen exposure, lymphocyte activation, clonal expansion, somatic hypermutation, and class-switch recombination further reshape immune repertoires, ultimately generating highly complex and dynamic BCR and TCR landscapes (Dunn-Walters et al. 2018). In the present study, we established cross-tissue single-cell profiling integrating paired BCR and TCR repertoires in healthy DLY crossbred piglets. By simultaneously profiling transcriptomes and adaptive immune receptors across 11 immune-associated tissues, this work provides a systematic view of the baseline organization of the porcine adaptive immune system under physiological conditions. Distinct immune cell compositions and adaptive immune architectures were observed among lymphoid, mucosal, and circulatory tissues, demonstrating substantial spatial heterogeneity of porcine immunity. Compared with previous porcine immune repertoire studies that mainly focused on bulk sequencing, individual tissues, infection models, or single receptor types (Wiarda et al. 2026), our study comprehensively resolves both humoral and cellular immune repertoires at single-cell resolution across multiple organs. Moreover, we establish a valuable resource for future studies of disease resistance in locally adapted commercial pig populations.

Our study also provides characterizations of porcine B-cell and T-cell heterogeneity. Based on some classic annotated genes (Herrera-Uribe et al. 2021), reclustering analyses identified multiple transcriptionally distinct adaptive immune subsets, including naive, activated, proliferative, cytotoxic, and memory-associated populations (Fig. 3A, C). The detection of cycling plasma cells and activated cytotoxic T-cell subsets in healthy animals suggests that immune turnover and low-level immune activation persist even under physiological conditions. Similar conclusions have also been found in other reports (Silva et al. 2016). Moreover, the strong enrichment of double-positive *CD4* + *CD8* + T cells within thymic tissues further reflects conserved developmental characteristics of porcine T-cell maturation (Fig. 3D), conforming to the reports of relevant literature (Schad et al. 2022). Together, these findings highlight the dynamic and continuously regulated nature of the healthy porcine immune system.

At the immune repertoire level, we observed highly structured and biased V(D)J gene usage patterns in both BCR and TCR repertoires. Several V and J gene segments were consistently enriched across tissues, indicating that receptor recombination in pigs follows preferential selection patterns rather than purely stochastic assembly (Fig. 4G, FigS. 3 A, B). This is also reflected in the research conducted by others (Schwartz and Murtaugh 2018). Compared with TCR repertoires, BCR repertoires exhibited stronger V–J pairing preferences and more frequent inter-tissue sharing. The mechanistic basis for this distinction remains to be fully elucidated. One plausible explanation involves the well-documented differential migratory capacities between lymphocyte subsets: B cells routinely recirculate through the blood and lymphatic systems (Pelissier et al. 2023), whereas T cells display a stronger propensity for tissue residency (Burton et al. 2024; Scott et al. 2024). However, our current transcriptional analysis of canonical homing receptors (such as CCR7, CXCR5, and CCR9) did not reveal a statistically significant difference between B and T cells in this dataset. This raises the possibility that the observed clonal sharing in B cells may be less dependent on differential chemokine receptor expression per se, and might instead involve alternative mechanisms—including, but not limited to, the inherently lower antigen-binding specificity of BCRs, which could promote convergent structural adaptations and thus facilitate clonal lineage sharing (Silver et al. 2017). Our comparative analysis revealed partial conservation of V/J gene usage patterns between the porcine and human adaptive immune repertoires, particularly for TCRs, while also highlighting substantial species-specific features. Several porcine TCR V/J genes, including TRAV21 and TRBV20-1, exhibited relatively high usage frequencies in our dataset and showed comparable utilization patterns in human TCR repertoires. In contrast, we did not observe clear concordance in BCR V/J gene usage between pigs and humans. These findings suggest that TCR V/J gene usage may retain some conserved features across species, whereas BCR V/J gene usage appears to be more species-specific. Representative cross-species V/J gene comparisons are summarized in Table 1 (Kitaura et al. 2016; Hong et al. 2018). Another important finding of this work is the extensive diversity yet relative stability of adaptive immune repertoires under healthy physiological conditions. Most clonotypes were represented by singleton or low-frequency clones, whereas highly expanded clonotypes occupied only a small fraction of the total repertoire (Fig. 5E, F). Such repertoire structures confirm the need to maintain broad antigen-recognition potential in the absence of acute immune challenge (Briney et al. 2019; Berruezo-Llacuna et al. 2025). Meanwhile, differences in diversity indices among tissues indicate that local immune environments shape repertoire richness and expansion dynamics differently (FigS. 4 C, D). Cross-tissue overlap analyses further revealed a complex balance between immune circulation and tissue specialization. Although most clonotypes exhibited tissue-restricted distributions, a few high-expansion-level BCR and TCR clonotypes were shared among multiple organs, especially among lymphoid and mucosal tissues (Fig. 5C, D). Given that all samples were collected under immune baseline conditions (without deliberate antigen challenge), the observed sharing is unlikely to reflect antigen-driven clonal expansion. Instead, we interpret this multi-organ sharing as a manifestation of the intrinsic V(D)J recombination bias—namely, that certain V/J gene combinations and CDR3 lengths are preferentially generated by the recombination machinery, resulting in the systemic coexistence of these 'public-like' clonotypes across tissues. This baseline preference provides a fundamental reference for understanding tissue-distributed immune repertoires under steady-state conditions (Buggert et al. 2020; Sureshchandra et al. 2024). Interestingly, BCR clonotypes demonstrated substantially stronger cross-tissue overlap than TCR clonotypes (Fig. 6A), whereas TCR repertoires appeared relatively tissue-constrained. This difference indicates distinct Immune recombination specificity between B cells and T cells in pigs.

**Table 1:** Several genes with similar expression patterns. Representative V/J genes were selected based on their relatively high usage frequencies in the porcine repertoire and the availability of corresponding human orthologs and published human repertoire data

Pig gene	Human ortholog	Gene type	Pig usage	Human usage
TRAV21	TRAV21	TRAV	5.8%	5.1%
TRAV9	TRAV9-1/2	TRAV	4.9%	11.2%
TRAJ43	TRAJ43	TRAJ	3.9%	2.1%
TRAJ40	TRAJ40	TRAJ	3.8%	2.0%
TRAJ37	TRAJ37	TRAJ	3.2%	7.5%
TRBV20-1	TRBV20-1	TRBV	7.7%	10.4%
TRBV15	TRBV15	TRBV	5.0%	4.5%
TRBJ1-1	TRBJ1-1	TRBJ	17.3%	7.8%
TRBJ1-2	TRBJ1-2	TRBJ	14.9%	7.5%
TRBJ2-3	TRBJ2-3	TRBJ	12.3%	10.1%

Clonotype definition can influence the estimation of repertoire diversity and clonotype sharing. In the related studies on immune repertoire, both the definition based on CDR3 amino acid sequence (Lepore et al. 2014) and the definition based on V-J-CDR3 have been adopted, with the latter providing a more stringent definition by additionally considering V and J gene usage. In the present study, we therefore compared the repertoire results obtained using these two definitions to evaluate the potential impact of our CDR3-based clonotype assignment (FigS. 5 A-F). As expected, defining clonotypes based on the V-J-CDR3 amino acid sequence resulted in a slight increase in the number of unique clonotypes. More notably, compared with the CDR3-only definition, the extent of clonotype sharing across tissues and cell types was slightly reduced. This difference is consistent with the possibility that identical CDR3 sequences may arise from distinct V/J gene rearrangements through convergent recombination. However, the magnitude of this effect was relatively small in our dataset. The overall patterns of clonotype abundance and distribution, as well as the major tissue- and cell-type-specific clonotype-sharing patterns, were highly consistent between the two definitions. Thus, although the CDR3-only definition may modestly overestimate clonotype sharing, this effect was limited and did not substantially affect the major conclusions of this study. These results also indicate that our repertoire findings are robust to the choice of clonotype definition.

A major strength of this study is the integration of single-cell transcriptomic profiling with paired TCR/BCR V(D)J sequencing, enabling simultaneous characterization of immune cell identities, functional states, and antigen receptor features at single-cell resolution. The integration of clonotype information with single-cell transcriptomes additionally enabled direct visualization of clonal expansion states within defined immune cell subsets (Fig. 6D, G). This integrative strategy allows immune dynamics to be interpreted not only at the repertoire level but also within precise cellular contexts, which is difficult to achieve using conventional bulk repertoire sequencing methods (Wu et al. 2024). Beyond repertoire structure, our analysis of immunoglobulin chain composition also revealed substantial heterogeneity in antibody usage among B-cell subsets. Differential utilization of IGK and IGL light chains (Fig. 7E), together with variable distributions of IGHM, IGHD, IGHA, and IGHG isotypes, reflects dynamic regulation of antibody maturation and class-switch recombination in healthy pigs (Fig. 7F). Variations in their relative usage proportions play a critical role in both the development of the immune system and the manifestation of favorable phenotypic traits (Stepanova et al. 2022; Qiu et al. 2024). Furthermore, the diversity of IGHG subclass usage suggests additional complexity within porcine humoral immunity (Fig. 7G), which has also been verified by previous works (Butler et al. 2009; Zhang et al. 2020). An important technical contribution to this study is the improved detection sensitivity achieved by our sequencing strategy (Fig. 7H). Compared with previously published porcine immune repertoire datasets, we identified larger numbers of immunoglobulin alleles and receptor combinations. This improvement results from the utilization of the Pig-specific V(D)J primers we designed, enabling us to more accurately capture the information of the immune repertoire and receptor pairing in single-cell samples (Gu et al. 2025). The enhanced coverage achieved in this study provides a more complete representation of porcine adaptive immune diversity and demonstrates the advantages of single-cell immune repertoire technologies over conventional repertoire sequencing approaches. This optimized amplification strategy represents a valuable tool for future single-cell immune repertoire studies and may contribute to more accurate characterization of antibody diversity in pigs.

Several limitations should nevertheless be considered. First, as a pooled-sample atlas, this study is not designed to assess inter-individual variability or to enable statistical inference at the population level. The primary goal is to provide a broad, high-resolution cellular inventory of the immune landscape across multiple tissues. We acknowledge that the pooling strategy, while enabling efficient and deep profiling of major cell types across tissues, precludes any assessment of biological reproducibility or differential abundance between individuals. Therefore, the generalizability of the observed cellular features in our research results still needs to be further verified in independent, non-pooled cohorts. Second, our cell-type annotations were primarily based on transcriptional profiles and canonical marker genes, without independent orthogonal validation. Although the identified marker expression patterns were consistent with the assigned cell identities, the relatively limited availability of well-validated porcine cell-type markers may introduce uncertainty, particularly for closely related immune cell subsets. Future studies should incorporate orthogonal approaches, such as flow cytometry, immunohistochemistry, or immunofluorescence, to independently validate major immune cell populations and their tissue distributions. Third, although extensive clonotype sharing and expansion patterns were identified, antigen specificity and functional receptor activity were not directly assessed. Fourth, the relatively limited sample size may underestimate rare clonotypes or low-frequency immune populations. Overall, this study establishes comprehensive cross-tissue single-cell profiling integrating both BCR and TCR repertoires in healthy DLY crossbred piglets. Our findings provide novel insights into the spatial organization, clonal architecture, and cellular heterogeneity of the DLY crossbred piglet adaptive immune system. As a foundational reference resource, this profiling will support future investigations of infectious diseases, vaccine responses, immune development, and translational immunology in pigs and other large-animal models.

## Supplementary Information

Supplementary Material 1.

Supplementary Material 2.

Supplementary Material 3.

Supplementary Material 4.

Supplementary Material 5.

Supplementary Material 6.

## Data Availability

The raw sequence data reported in this paper have been deposited in the Genome Sequence Archive (Genomics, Proteomics & Bioinformatics 2025) in National Genomics Data Center (Nucleic Acids Res 2025), China National Center for Bioinformation/Beijing Institute of Genomics, Chinese Academy of Sciences (GSA: CRA043620) that are publicly accessible at https://ngdc.cncb.ac.cn/gsa.
