# Supplementary material for: A single-cell profile of the porcine immune repertoire: concurrent profiling of T cell receptor and B cell receptor V(D)J sequences in health
Source: Adv Biotechnol (Singap). 2026 Oct 9;4(4):38. doi: 10.1007/s44307-026-00139-2 (PMC13650219; doi:10.1007/s44307-026-00139-2)
Supplement: Supplementary file 1 [file 44307_2026_139_MOESM1_ESM.docx]

**FigS. 1** **A** The distribution of three QC metrics (nCount_RNA, nFeature_RNA, Percent.mt); the red dotted line indicates the QC threshold. **B** The number of cells before (pre) and after (after) quality filtering for each sample. **C** Comparison of the cell umap plots before and after batch effect removal. Different organizations use different colors for marking, and the LISI method is employed for quantitative comparison to assess the treatment effects.

**FigS. 2 A**-**D** Frequency distribution of V gene usage in BCR heavy chains, BCR light chains, TCR α chains, and TCR β chains. **E**-**H** Frequency distribution of J gene usage in BCR heavy chains, BCR light chains, TCR α chains, and TCR β chains.

**FigS. 3 A**-**B** V–J pairing frequencies of TCR α chains and β chains.

**FigS. 4 A-B** Clonotype abundance distribution curves of BCR and TCR repertoires. **C-D** Diversity analysis of BCR and TCR repertoires based on Shannon index, inverse Simpson index, ACE index, and Chao1 index scores.

**FigS. 5 A-B** Numbers of unique BCR and TCR clonotypes across tissues, respectively. **C-D** Numbers of unique BCR and TCR clonotypes across cell types, respectively. Numbers shown in black indicate clonotypes defined by the combined V–J–CDR3 sequence, whereas numbers in red parentheses indicate clonotypes defined solely by identical CDR3 amino acid sequences. **E-F** Heatmaps showed BCR and TCR clonotype sharing across tissues under the two clonotype definitions. Compared with the CDR3-only definition, the stringent V–J–CDR3 definition resulted in modest reductions in shared clonotypes, while preserving the overall tissue- and cell-type-specific repertoire patterns.
